# On the way to the azygos vein: a road of return rather than ruined

**DOI:** 10.1186/s13019-024-02708-9

**Published:** 2024-04-20

**Authors:** Yiping Feng, Yeqing Liu, Shanxiang Xu, Huiming Zhong, Shouyin Jiang

**Affiliations:** 1 Department of Emergency Medicine, Second Affiliated Hospital, Zhejiang University School of Medicine, Hangzhou, 310009 China; 2 Department of Pathology, The Children’s Hospital, Zhejiang University School of Medicine, National Clinical Research Center for Child Heath, Hangzhou, China; 3 Key Laboratory of The Diagnosis and Treatment of Severe Trauma and Burn of Zhejiang Province; Zhejiang Province Clinical Research Center for Emergency and Critical Care Medicine; Research Institute of Emergency Medicine, Zhejiang University, Hangzhou, 310009 China

**Keywords:** Central venous catheter, Malposition, Azygos vein

## Abstract

**Background:**

The malposition of central venous catheters (CVCs) may lead to vascular damage, perforation, and even mediastinal injury. The malposition of CVC from the right subclavian vein into the azygos vein is extremely rare. Here, we report a patient with CVC malposition into the azygos vein via the right subclavian vein. We conduct a comprehensive review of the anatomical structure of the azygos vein and the manifestations associated with azygos vein malposition. Additionally, we explore the resolution of repositioning the catheter into the superior vena cava by carefully withdrawing a specific length of the catheter.

**Case presentation:**

A 79-year-old female presented to our department with symptoms of complete intestinal obstruction. A double-lumen CVC was inserted via the right subclavian vein to facilitate total parenteral nutrition. Due to the slow onset of sedative medications during surgery, the anesthetist erroneously believed that the CVC had penetrated the superior vena cava, leading to the premature removal of the CVC. Postoperative contrast-enhanced computed tomography of the chest confirmed that the central venous catheter had not penetrated the superior vena cava but malpositioned into the azygos vein. The patient was discharged 15 days after surgery without any complications.

**Conclusions:**

CVC malposition into the azygos vein is extremely rare. Clinical practitioners should be vigilant regarding this form of catheter misplacement. Ensuring the accurate positioning of the CVC before each infusion is crucial. Utilizing chest X-rays in both frontal and lateral views, as well as chest computed tomography, can aid in confirming the presence of catheter misplacement.

## Introduction

Central venous catheters (CVCs) have been widely used in clinical practice to meet the infusion needs of patients requiring administration of irritant drugs, total parenteral nutrition (TPN), or when peripheral venous access is limited [1]. Among various insertion sites, placement of CVC through the right subclavian vein (RSV) is one of the most commonly used approaches. While CVC is often a necessary procedure for treatment, it is not without risks. Potential complications may include pneumothorax, vascular puncture, and even mediastinal injury [2]. CVC malposition is not a complication, and its occurrence is significantly associated with complications such as thrombosis and vascular injury [3]. In clinical practice, the conventional approach involves the removal and replacement of the catheter. However, it is important to acknowledge that the replacement of the catheter carries inherent risks, including the potential for invasive surgical injuries and subsequent complications.

Here, we report a rare case of CVC malposition into the azygos vein (AV) via RSV. The anesthesiologist mistakenly believed that the catheter had perforated the vessel wall and prematurely removed it. The malpositioned catheter could be adjusted and continued to be used after timely repositioning, thus avoiding unnecessary removal and reinsertion of the catheter.

## Case presentation

A 79-year-old female presented to our department with symptoms of complete intestinal obstruction. The patient initially presented with symptoms of abdominal distension and abdominal pain ten days prior, with a temporary amelioration following laxative administration. However, six days thereafter, the patient’s condition deteriorated, marked by escalating abdominal pain, persistent distension, and a conspicuous cessation of gas and bowel movements, culminating in her hospitalization. Clinical examination disclosed pronounced abdominal swelling with tenderness but the absence of rebound tenderness.

An urgent abdominal computed tomography (CT) scan revealed the presence of a colonic tumor and concomitant complete intestinal obstruction. The patient’s treatment regimen encompassed fasting and the administration of gastric decompression therapy. The patient’s documented history of reduced oral intake and a computed Nutrition Risk Screening 2002 score of 3 were indicative of a need for TPN.

The emergency physician executed a RSV puncture to facilitate central venous catheterization. The puncture needle was inserted into the RSV, resulting in the acquisition of nonpulsatile blood flow. Subsequently, a double-lumen catheter (8 French, Flex Tip, 20 cm length, Arrow) was meticulously advanced to a depth of 15 cm within the RSV, under the precise guidance of a J-tip guide wire. Both lumens were readily aspirated and flushed with a saline solution.

Following the successful procedure, the patient initiated TPN at a rate of 1720 ml/day, with a nonprotein caloric intake of 970 kcal/day through intravenous infusion. The infusion process transpired without any discernible complications or discomfort to the patient.

**Fig. 1:**
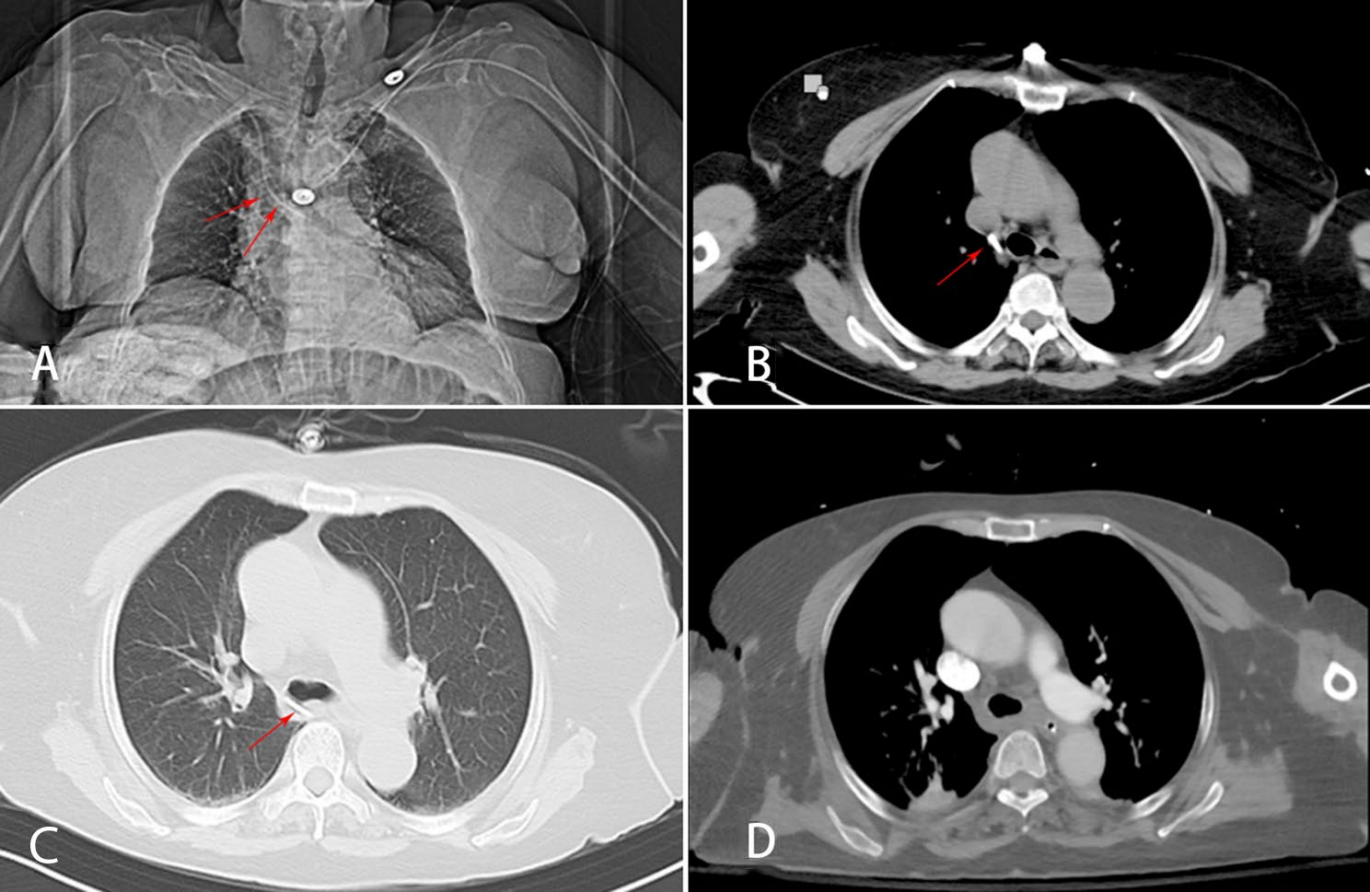
(**A**) Chest radiograph showing a central venous catheter inserted via the right subclavian vein and the catheter kinking at the right tracheobronchial angle (red arrows); (**B**) Axial slice from the chest CT scan shows the catheter tip projecting from the posterior wall of the superior vena cava (red arrow); (**C**) The CVC deviated anteriorly towards the thoracic vertebrae (red arrow); (**D**) Post-operative contrast-enhanced chest CT revealed no pleural effusion or mediastinal fluid accumulation

Two days after admission, the patient underwent exploratory laparotomy and radical resection surgery for the sigmoid colon tumor. Due to the prevalence of COVID-19, patients undergoing general anesthesia for surgery in our hospital are routinely subjected to preoperative chest CT scans to ascertain the concurrence of COVID-19. However, prior to the surgery, we failed to timely detect the anomalous trajectory of the CVC from the chest CT (Fig. 1A). Anesthesia induction was accomplished using etomidate (0.18 mg/kg), rocuronium (0.75 mg/kg), and sufentanil (0.67 µg/kg) administered via the CVC, concurrently with the inhalation of sevoflurane. However, the sedation effect was suboptimal. The anesthetist, upon reviewing the patient’s chest CT scan, observed that the tip of the CVC appeared to have possibly protruded out of the vascular wall through the posterior aspect of the superior vena cava(SVC) (Fig. 1B). At that time, there were concerns that the catheter may have breached the vascular wall and entered the mediastinum, leading to the decision to remove the deep venous catheter and reposition a CVC via the right femoral vein.

**Fig. 2:**
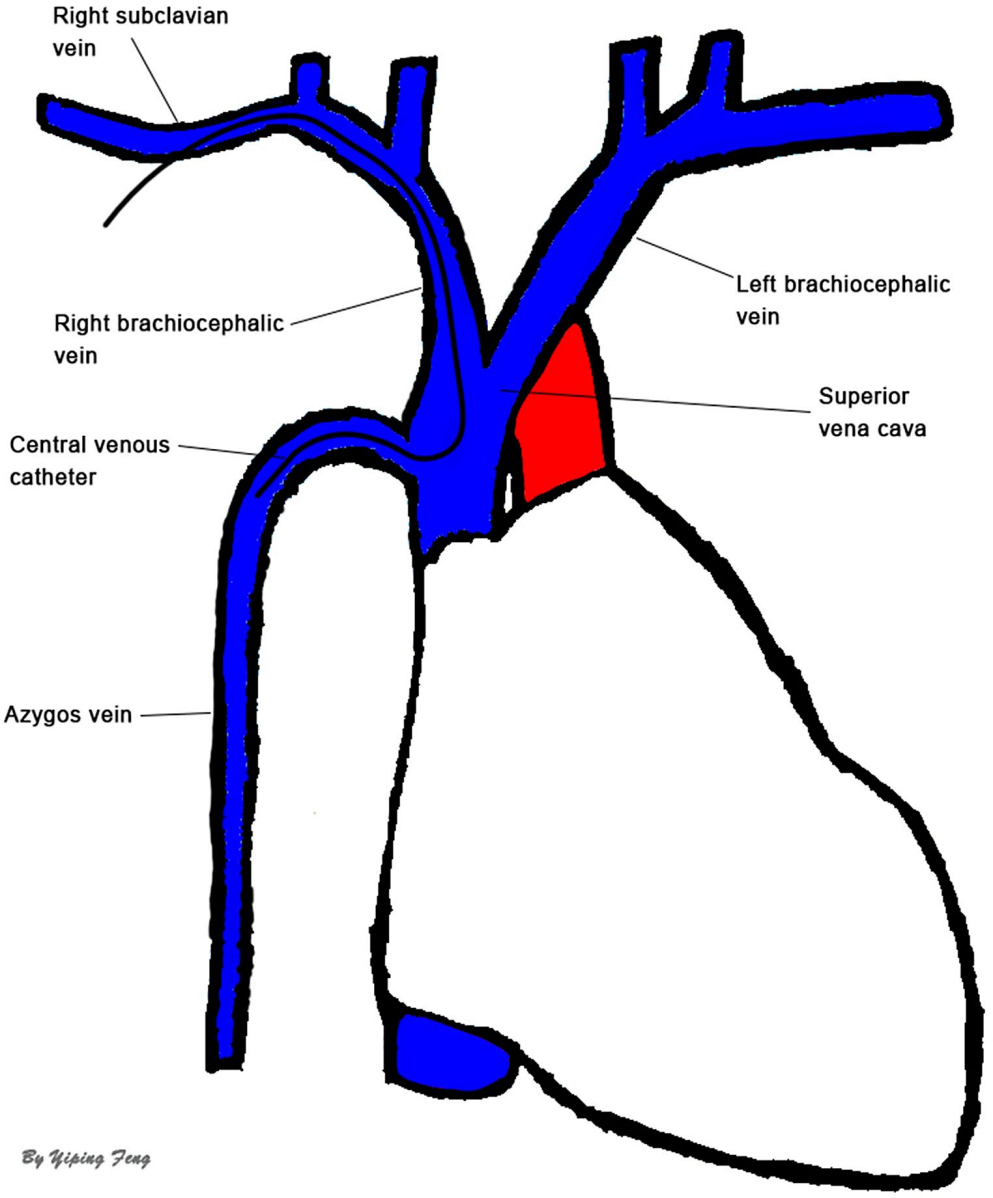
Anatomic illustration showing the course of the azygos vein and the aberrant trajectory of the central venous catheter

After the patient’s surgery, we meticulously reviewed the patient’s chest CT and observed that the CVC, upon leaving the superior vena cava, deviated anteriorly towards the thoracic vertebrae. This trajectory corresponded to the course of the AV, which ascended anteriorly from T12 to T5 before joining the SVC (Fig. 1C). On the day following the patient’s surgical procedure, a comprehensive enhanced chest CT scan did not reveal any pleural or mediastinal effusion (Fig. 1D). Combining anatomical and contrast-enhanced CT imaging, it was considered that the CVC had taken an anomalous route into the AV without piercing any blood vessels (Fig. 2). The patient had a good recovery and was discharged in improved condition after 15 days.

## Discussion

The AV, a branch of the SVC, originates from the right ascending lumbar vein. It ascends along the right side of the thoracic vertebra, gently curves forward around the 4th or 5th thoracic vertebra level, and ultimately drains into the SVC at its posterior wall [4]. Prior to its confluence with the right brachiocephalic vein, which gives rise to the SVC, the left brachiocephalic vein follows a trajectory from anterior to posterior. Consequently, when a CVC is introduced through the left side, it has a higher likelihood of entering the AV, owing to the reduced angle between the left brachiocephalic vein and the point of entry to the AV, in comparison to insertion from the right side. Previous research has also indicated that most catheter malpositions into the AV were inserted from the left side [5]. Furthermore, due to the narrower diameter of peripherally inserted central venous catheters (PICCs), the occurrence of misplacement is more common, while it is relatively less frequent with CVCs. We have not found any recent reports about unintentional AV insertion caused by CVC placement through RSV.

The risk factors for venous catheter malposition into the AV include left-sided catheterization, PICC placement, smaller catheter diameters, insufficient catheter insertion depth, and high intrathoracic or intraabdominal pressures [6]. In this case, the patient suffered from complete intestinal obstruction due to a colonic tumor, resulting in an increase in intra-abdominal pressure. This may have been the cause of the malposition of the CVC into the AV.

Assessment of the function of CVC before catheter use is the basis for the safety of CVC use, and blood withdrawal from the CVC is the primary method. Failure in blood withdrawals may indicate malposition or fold-back of the CVC tip, and further analysis is necessary. When CVC malposition is suspected, an ultrasound examination can be performed to determine whether the catheter is within the subclavian vein and to check for malposition into the internal jugular vein, which is the most frequent type of misplacement [7].

In cases where CVC malposition into the AV is suspected, a chest radiograph in the anteroposterior view or a chest CT scan is needed. On a chest radiograph, malposition into the AV can manifest as kinking of the guidewire or catheter at the right tracheobronchial angle [8] (Fig. 1A). The tracheobronchial angle, the “corner” at which the trachea gives off the right main bronchus, is a constant anatomic landmark on frontal radiography and marks the exact location where the AV arches over the right main bronchus to enter the SVC. A lateral chest radiograph can display the deep vein catheter extending posteriorly at the level of the 4th to 5th thoracic vertebra. A chest CT scan allows for a more precise observation of the position of the CVC catheter tip.

When CVC malposition into the AV, it can form a right or acute angle. Due to the relatively smaller diameter of the AV, measuring approximately 0.96 ± 0.18 cm, CVC that misplaces into the AV may mechanically stimulate the vessel wall during the process of infusion, potentially leading to complications such as catheter occlusion, thrombus formation, vascular wall damage, or perforation [2, 9]. In addition, in the intensive care unit (ICU) setting, fluid resuscitation or infusion of vasoactive drugs may increase the risk of vascular injury after CVC into the AV. Interestingly, the central venous pressure (CVP) measured at AV is higher than the CVP at SVC [10]. An elevated CVP inconsistent with a patient in shock could be a clue of catheter malposition. To our knowledge, CVC malpositioning into AV through RSV is rare and could be missed. Clinicians should be aware of such a potential risk.

There are two solutions to AV malpositions: catheter removal or adjustment of the CVC. If the CVC remains unobstructed and the patient exhibits no significant symptoms, the extent of misplacement can be assessed using a chest X-ray or chest CT. By carefully withdrawing the CVC to the corresponding length, it can be repositioned into the SVC. In cases where CVC obstruction or symptoms such as chest tightness, chest pain, or pleural effusion arise, it is advisable to conduct an enhanced chest CT to determine the presence of vascular perforation. In the event of vascular perforation, it is recommended to leave the CVC in place and seek a vascular surgery consultation to assess the requirement for interventional radiology treatment. Hasty removal of the CVC may lead to substantial hemorrhage.

## Conclusion

It is critical to determine the true position of the CVC before every infusion. When there is difficulty in either infusing or aspirating through the CVC, or if there is a delayed onset of drug action, the possibility of catheter malposition into the AV should be considered. Chest X-rays in both frontal and lateral views, as well as chest CT, can assist in confirming the presence of catheter misplacement.

## Data Availability

The datasets used and/or analyzed during the current study are available from thecorresponding author on reasonable request.
